# Indestructible plastic: the neuroscience of the new aging brain

**DOI:** 10.3389/fnhum.2014.00219

**Published:** 2014-04-11

**Authors:** Constance Holman, Etienne de Villers-Sidani

**Affiliations:** ^1^Medical Neurosciences Program, Charité UniversitätsmedizinBerlin, Germany; ^2^Montreal Neurological Institute, McGill UniversityMontreal, Canada

**Keywords:** plasticity, aging, cognitive training, computerized training, neurotechnology, cognitive decline, normality, biocapital

## Abstract

In recent years, research on experience-dependent plasticity has provided valuable insight on adaptation to environmental input across the lifespan, and advances in understanding the minute cellular changes underlying the brain’s capacity for self-reorganization have opened exciting new possibilities for treating illness and injury. Ongoing work in this line of inquiry has also come to deeply influence another field: cognitive neuroscience of the normal aging. This complex process, once considered inevitable or beyond the reach of treatment, has been transformed into an arena of intense investigation and strategic intervention. However, important questions remain about this characterization of the aging brain, and the assumptions it makes about the social, cultural, and biological space occupied by cognition in the older individual and body. The following paper will provide a critical examination of the move from basic experiments on the neurophysiology of experience-dependent plasticity to the growing market for (and public conception of) cognitive aging as a medicalized space for intervention by neuroscience-backed technologies. Entangled with changing concepts of normality, pathology, and self-preservation, we will argue that this new understanding, led by personalized cognitive training strategies, is approaching a point where interdisciplinary research is crucial to provide a holistic and nuanced understanding of the aging process. This new outlook will allow us to move forward in a space where our knowledge, like our new conception of the brain, is never static.

## A brief history of brain plasticity

The human brain is the basis for many metaphors, qualifying everything from how we succumb to reflex and addiction to explaining how personality and selfhood is maintained over a lifetime (Dumit, 2004; Wexler, 2006; Lock, 2013). Yet within this anchoring framework, change is never-ceasing, encompassed by the vast range of neurological modifications known collectively as neuroplasticity. As a concept, the changeable brain is an immensely fuzzy entity, and plasticity can include everything from microscopic modifications of proteins at the synapse or incorporation of new neurons, to large-scale overhaul of cortical and subcortical areas in response to injury, illness or concerted learning. Far from occurring only in adaptive or reparatory circumstances, plasticity is also entwined with numerous disease processes, promoting cascades of maladaptive signaling patterns or abnormal morphology. This homogenous collection of modifications has never been party to a sole method of study or understanding in the neurosciences, and even today, scientists who study plasticity have dramatically different ideas about its range, role in day-to-day brain function and potential for use (Kempermann, 2006; Wexler, 2006; Rees, 2010). However, most can agree that it represents a challenging way to understand the nervous system, and as such, is often incompletely interpreted in descriptions of the brain and mind.

When the human brain is spoken of in terms of malleability, experience-dependent plasticity, or the capacity of the brain to alter itself in response to environmental input, is most often implicated. The fundamental morphological changes of this process, first hypothesized by early neuroscientists such as Cajal at the synaptic level (DeFelipe, 2006), are today understood to occur at most levels of organization in the nervous system. Plasticity is a fundamental part of the brain’s complexity, allowing it to adapt to subtle environmental changes on the timescale of milliseconds, and maintain the most useful of these modifications for a lifetime. Yet far from a universal process, plasticity of this type was thought to be segregated into distinct periods within an individual’s life. From experiments in the 1960s onward, the vast majority of the brain’s capacity for flexibility was understood to occur early in the lifecourse, preparing an organism for salient environmental cues, and “locking in” the necessary skills and behaviors to assure continued survival and thriving. Large-scale changes and learning were limited to discrete “critical periods”, after which the brain could not be significantly altered. This version of the moldable, adaptable brain has been adopted in a number of fields, most notably, developmental neurolinguistics (cf. Penfield and Roberts, 1959; Lenneberg, 1967; Wexler, 2006).

However, a broader, more nuanced version of this framework has recently begun to creep into public discourse, elaborating on the possibilities for large-scale change *outside* of embryogenesis or childhood. No-one believes that adults cannot learn, but it is fairly universally acknowledged that the process is slower, more laborious, and perhaps ultimately less efficient than is the case for younger brains. Only in truly exceptional experiences, such as after large-scale damage to the brain, can individuals seemingly break through boundaries encoded since childhood to make significant changes in brain wiring and patterns of connectivity. Just such exceptional circumstances provided early experimental frameworks for exploring the boundaries of plasticity in adulthood. In the mid-1970s, it was shown that using adult non-human primates and dramatic cessation of peripheral input (in perhaps the most notable instance, via finger amputation) could change allocation of space on the brain’s somatosensory cortex (Kaas et al., 1983; Merzenich et al., 1984). Though experimental manipulations were fairly crude by today’s standards, the amount of change elicited in the brain was truly remarkable, exceeding expectations, and resulting in complex changes at a cortical, subcortical, and even midbrain-level (Wall et al., 2002).

Despite the exciting findings of this early work, adult neuroplasticity had to gain a more tangible footing in everyday experience to attain the widespread recognition and seriousness previously accorded to well-known facts in neuroscientific discourse (Hacking, 1995, 1999; Young, 1995). This recognition arrived with three major advances in the 1990s. First, a landmark series of studies in birds, rodents and higher mammals solidified the concept of neural genesis in the adult brain, widening conversations and debates about learning and adaptability beyond traditional critical periods (Ming and Song, 2005; Rubin, 2009; Rees, 2010). Secondly, through continuing work with primates, the notion of experience-dependent plasticity could be quantified on much more subtle (and less dangerous) terms, showing that as a result of *learning*, the brain could be modified (Rencanzone et al., 1993; Xerri et al., 1998). Building on these studies, improved neuroimaging technology further allowed neuroscientists to look for evidence of structural experienced-based changes in the human brain. Through work with diverse experimental paradigms, they found them, exceeding the wildest expectations of the scientific community only a decade before. Employment, musical training, and even studying for exams appeared to be sufficient to promote large-scale structural changes in the brain outside of childhood (Maguire et al., 2000; Gaser and Schlaug, 2005; Draganski et al., 2006; Draganski and May, 2008). No longer could the adult brain be considered a fixed entity, immune to the mundane activities of day-to-day life, and impervious to new learning or restructuring.

This level of malleability challenges neurobiologists struggling to pin down its biochemical substrates as well as decades of discourse and knowledge about the relationship between an individual, the brain, the mind and the environment (Malabou, 2008). However, nowhere do these ideas about change and flexibility have more potential to overhaul common understanding and dialogue than in the study of the aging process (Baltes et al., 2006; Katz and Peters, 2008; Williams et al., 2012). Older adults have long occupied a strange zone of tension and liminality, exemplifying the view of adult-brain-as-fixed-entity, and straddling the line between retention of past experiences and loss of “self” through pathology (Baltes and Smith, 2003; Corner and Bond, 2004). Challenging decades of crystallized knowledge about a static or deteriorating brain is a complicated enterprise, yet the notion of a flexible older brain is moving into the public mind with remarkable swiftness, borne further along with every advance in the neuroscience of adult plasticity (Katz and Peters, 2008; Malabou, 2008; Rees, 2010). What has enabled this evolution, and how has neuroscience been transformed into a discussion of lifestyle, new self-defined aging and re-definition of its boundaries? To answer these questions, the many understandings of aging today need to be disentangled, tracing back the current state of affairs to a complex negotiation and renegotiation of the aging mind and brain in biomedical discourse.

### Neuroscientific encounters with aging

Of course, it cannot be said that the aging brain was always an object of concerted inquiry. Like many other fields, it has been deeply influenced by interplay of scientific and cultural forces during its development (cf. Hacking, 1999; Knorr-Cetina, 1999). Since the earliest treatises on the human body, individuals have recognized that advancing age engenders numerous changes in thought and action, leading to a gradual slowing of cognitive functions and adaptability to the environment. Earlyt scholars ascribed these changes to complex environmental interactions and processes, and it was not until the late 19th century that technological advances in microscopy and formalized neurology allowed doctors such as Charcot to make more firm divisions between what was pathological, and what was expected as a part of more “normal” decline (Achenbaum, 1974; Katz, 1996, 2006). The psychological tradition was somewhat slower to begin describing the aging mind in its own right, but nonetheless early authors often had a few words to say on the topic. During this time, most literature on the topic described a sort of “rigidity” accompanying the aging process in the mind and brain, and even went so far as to describe the aging mind with “the cognitive grooves and channels set, the power of assimilation gone” (James, 1893). Even early gerontological literature such as G. Stanley Hall’s *Senescence* qualified older individuals as “battered, water-logged leaking derelicts”, and emphasized decay of mental faculties and adaptability (Hall, 1922; Cole, 1984). The aging mind, unfortunately, was also excluded from much early psychoanalytic study. In the words of Sigmund Freud (1905) “Near and above the fifties, the elasticity of the mental processes on which [psychoanalytic therapy] depends, is, as a rule, lacking- old people are no longer educable…”. This (admittedly somewhat dismal) outlook shaped the concept of aging as of one of passive, inevitable decline, where if one was lucky enough to avoid pathological changes, slow, progressive decline of mental flexibility and adaptability could be expected with time.

Happily, today this type of thinking has seen an about-face, and aging is seen in a far less “restrictive” manner. This change has been enabled by numerous factors, most centrally, a move to study aging individuals and their brains in the absence of pathology. In turn, this line of thought has severely undermined the belief that the aging brain is set in stone, and grounded entire lines of inquiry in examining late-life change and adaptability. While in large part, development of improved experimental paradigms and neuroimaging techniques may account for this latter interest, the changeable, environmentally-sensitive brain may in fact be located much earlier, coinciding with more sophisticated understandings of life in old age brought about by a rising interest in social welfare early in the 20th century (Hirshbein, 2000; Pickard, 2011).

With modernizing societies in Europe and North America at this time, formalized gerontology emerged in the 1910s and 20s with a view of defining more rigorous standards for shaping normality in aging and partitioning which groups of seniors should be party to health interventions (cf. Lorge, 1948; Sheldon, 1948; Pickard, 2011). Wide epidemiological surveys (cf. Terman, 1916; Jones and Conrad, 1933) and the beginning of development of formalized cognitive testing such as the Wechsler–Bellevue Adult Intelligence scales (Wechsler, 1939) further reinforced ideas about defining intelligence and other cognitive processes across the lifecourse (Schaie, 2005). Partly due to this new body of quantifiable knowledge, several investigators also began examining how environmental factors such as low socioeconomic status or lack of community/familial involvement could be correlated with poor cognitive or physical outcomes for seniors (Kuhlen, 1940; Sheldon, 1948; Townsend, 1957). In one sense, studying the deleterious effects of these factors could be seen to provide a strong grounding for experience-dependent plasticity in old age. However, apart from a newfound respect for environmental factors affecting individuals’ well-being later in life, and general programs aiming to improve (usually financial) wellbeing of seniors such as Social Security in the United States, cognitive decline was not tackled in a preventative, or changeable nature (Katz, 1996, 2006; Hendricks and Achenbaum, 1999).

The first interventions aimed at making permanent physical changes to the aging brain grew from studies of stroke rehabilitation in the early 20th century (cf. Warren, 1950; Blaikie, 1999; Carr and Shepherd, 2006). Drawing heavily on beliefs about reflexology and operant conditioning, investigators explored how discrete, repetitive activities could be used “*to restore* motor ability, insofar as is possible, within the irreversible limitations set by injury or disease, and the process of aging” (Voss, 1967, emphasis in original). Early rehabilitation for brain-based trauma had modest success, yet in many ways surpassed progress in understanding more subtle forms of age-related changes in cognition at that time. Despite technological advances permitting better understanding of pathological processes, the search for effective treatment or streamlined *in vivo* diagnostic markers for age-related cognitive decline remained fruitless. However, somewhat later, the first early studies in purely *cognitive* rehabilitation began to be performed (cf. Hoyer et al., 1973; Baltes and Baltes, 1977; Poon et al., 1980). Most investigators reported some measure of success in improving efficiency of cognitive processes, but their results failed to gain public notoriety. Today, it is difficult to explain why such promising findings that had potential to dramatically shift ideas about change in aging remained relegated to discrete circles of neuroscientific inquiry. One possibility is that their rubric of success, improved performance on neurocognitive tests, did not immediately suggest applicability to everyday problems faced by aging individuals with declining cognitive function. Another is that the investigations were just too early: unlike the widespread excitement about plasticity in younger adults brought about by advances in the 1990s, as well as a lack of visible changes made possible by later technological developments, cognitive training (CT) stayed quietly as an area of interest pursued by a small body of investigators in careful, but limited studies (Willis, 1990).

However, in the late 1990s and early 2000s, advances in *in vivo* imaging technology provided the impetus for the cognitive neuroscience of aging to gather momentum through a series of landmark studies. Not surprisingly, this time frame also coincided with a growing interest in the disease burden caused by the aging baby boom generation, which called governments worldwide to gain new understanding of citizens who would soon be using health care resources (particularly for conditions such as dementia) at an unprecedented rate (Brookmeyer et al., 2007; Moreira, 2009a). For the first time, aging adults defined as within “normal” ranges of function for their age cohort were subject to imaging as an object of inquiry, providing a more sensitive window to structural and metabolic correlates of slowing cognition in later life. This development coincided with the development of higher-resolution in imaging techniques, which was able to present several candidates for nuanced structural correlates of aging, primarily seen in evidence for changes in white- and gray-matter density over the lifecourse. (Raz et al., 2005; Batouli et al., 2014). Furthermore, it appeared that changes in volume in certain areas such as the frontal or dorso-lateral—prefrontal cortex, known to be implicated in a variety of high-level cognitive functions (Goldman-Rakic, 1995; Miller, 1999), could be correlated with poor performance on a variety of cognitive tasks (Raz et al., 1999; Rypma and D’Esposito, 2000; Bartzokis et al., 2001). Other intriguing findings came from studies of functional neuroimaging, where investigators first remarked that aged brains demonstrated dramatically different patterns of activation in response to cognitive testing than younger brains. Not only did it appear that blood-oxygenation level-dependent (BOLD) activity was more diffuse in the aged brain, but also that tasks normally lateralized to a single hemisphere spread to the other (Spreng et al., 2010; Eyler et al., 2011). The exact reasons for these changes is still debated (cf. Reuter-Lorenz and Park, 2010; Grady, 2012), but without a doubt, they have changed the types on conversation had about cognitive aging. No longer could declines in cognitive function be ascribed solely to decay/neurodegeneration, but instead are spoken about in terms such as “adaptation”, “compensation”, and “re-allocation”(Hazlett et al., 1998; Reuter-Lorenz and Cappell, 2008; Park and Reuter-Lorenz, 2009), framing the aging brain as having *agency* in a changing environment. Indeed, instead of gradually shutting down in the face of advancing years, the aging brain appeared to demonstrate impressive reorganization and plastic adaptation in its own right.

These developments in and of themselves modified conversations about the aging brain, but it would take one more wave of advances to catapult neuroplasticity into the realm of accessibility and malleability as discussed today. If the normally aging brain was modifiable to the extent that cognitive slowing could be correlated with discrete structural or “adaptive” metabolic changes, then could a mobilization of plasticity-inducing experimental paradigms be used to attenuate or reverse these changes? In younger brains, the evidence pointed toward yes, showing impressive experience/training-based reorganization (Draganski and May, 2008; Scholz et al., 2009; Kanai and Rees, 2011). Investigators such as Bäckman and May began to more closely examine training-based changes in cognition in the elderly using structural and functional imaging methods. The results were unexpectedly good, demonstrating that anywhere from several hours to 3 months of training on a given task could produce patterns of activation more similar to that seen in a younger person (Nyberg et al., 2003; Erikson et al., 2007), generalize to other domains (Berry et al., 2010), and even produce large structural changes in gray matter density and connectivity (Boyke et al., 2008). While still confined to a laboratory environment, it appeared that changes in age-related substrates could be effectively targeted, laying the framework for a new way of interacting with and modifying the aging brain.

## Conceptualizing cognitive training

Recognizing the potential of such findings to be implemented in a constructive fashion with aging populations worldwide, teams of scientists, investors and software developers began working together in the early 2000s to create sophisticated training programs that could be delivered to consumers in an electronic format. What, exactly, are these programs? Electronic CT is an immensely diverse field, but the basic premise of all programs relies on using specially-designed training to shape neural connectivity and efficiency underlying cognitive processes such as attention or working memory. Distinct from free online “brain training games” such as Sudoku, these programs rely on personalized measures of cognitive acuity such as reaction time, and tailor difficulty and duration of tasks to the user. While some more elaborate setups may include neural feedback devices, the most popular products on the market consist of a series of games based on (perhaps somewhat drier) validated training techniques from lab-based CT human. One of the most remarkable aspects of these programs is the diversity of their clientele. While some companies offer a general purpose program that allow “anyone to achieve their full potential” (Lumosity, 2013, “About Lumosity”), other more recent products have specifically developed platforms for individuals with everything from attention deficit hyperactive disorder (Pearson, 2014, “ADHD and Beyond”; PRweb, 2013, “CogniFit launches a specific brain training for ADHD”) to traumatic brain injuries (Eschen, 2012, “Brain Injury Survivor”). Although programs may be geared toward individuals with neuropathology, they are not formally recognized by any national diagnostic or prescriptive body as a formal sources of medical treatment. Aside from “retraining the brain” in the presence of a host of medical conditions, many sites emphasize how their products benefit healthy users too. Several sites contain extensive testimonials from individuals who have used their product to excel at work, school, and day-to-day tasks such as driving (cf. Posit Science, 2014, “Proven in lives”; Pearson, 2014, “User stories”; Vivity Labs, 2014, “What our users are saying”).

However, as this paper specifically examines CT’s relationship with aging, several criteria were used to identify companies that work specifically in this area. First, candidate CT companies were tracked-down using trade publications to identify companies with the largest customer base and revenue flow that sell training software to the public. Subsequently, these companies’ online literature was reviewed, and only groups who specifically mention their product targeting older adults. By this rubric, Lumosity (Lumos Labs), Brain HQ (Posit Neuroscience), Cogmed (Pearson), CogniFit (CogniFit), HappyNeuron (HAPPYneuron), Dakim Brain Fitness (Dakim), Fit Brains (Vivity Labs), and NeuroActive (Brain Center International) were all selected. From there, all product-associated information available on the internet, including advertisement, scientific claims and associated blogs were analyzed for information grouped into 4 clusters: (1) Neuroplasticity and its role in CT programming; (2) Definition of normal cognitive aging; (3) Explanation of age-related cognitive impairment and other pathology; and (4) Sociocultural factors involved in shaping brain health. The first cluster on CT mechanics will be explored in the following paragraphs, while subsequent themes correspond to later sections of this paper. Coverage of these issues naturally varied by site, but every effort has been made in this paper to provide themes common to several. This strategy, while providing a basic overview, does not represent a formal textual analysis and has several limitations which are discussed near the end of this paper.

While each of the CT programs evaluated differs slightly in layout and approach, all programs describe the science of experience-dependent plasticity as the foundation of their product. As one group explains, plasticity “the physical changes that are continually taking place in your brain as you experience and adapt to the world around you” (Lumosity, 2014, “How cognitive training works”). Other sites invoke similar metaphors, using new connectivity and neurogenesis to create an image of a strong brain, resistant to the onslaught of injury or pathology (cf. HAPPYneuron., 2013, “Efficiency of brain training”; Pearson, 2013, “Cogmed is based on a research breakthrough”; Pearson, 2013, “Brain fitness and brain training”). While the language is simplified, the basic idea holds relatively true to the neuroscience of plasticity. Using more recent findings, the consumer is further assured that this processes continues well into adult life. Here, the language becomes very empowerment-heavy, stressing how adult neuroplasticity has made a fundamental change in the way aging is perceived. “There really is no critical period of brain development unless one considers life itself to be the measure” claims one group, rather dubiously (Pearson, 2013, “Brain fitness and brain training”), while another states “brain plasticity is driving a revolution in brain health and science” (Posit Science, 2013, “Brain plasticity exercises”). In case there was any doubt in the mind of prospective consumers, it is also made very clear that these ideas extend to older individuals as well. Whether a company speaks of “empowering seniors” (Dakim, Inc, 2013, “The science behind Dakim Brain Fitness”) or “mak[ing] improvements at any age” (Pearson, 2013, “Brain fitness and brain training”), a clear link is made to using neuroplasticity-based training and shaping a healthier brain in later life.

All together, these groups are united by claims of “retraining” the aging brain by exploiting neuroplasiticty, with a view of preserving cognitive function into later life (cf. Dakim, Inc, 2013, “The science behind Dakim Brain Fitness”; Lumosity, 2013, “About Lumosity”; Posit Science, 2013, “Why should you use BrainHQ?”). Training strategies themselves differ from site to site, but many target cognitive functions with a variety of games exercising functions such as working memory, auditory precision, and processing speed. Many of the larger companies also devote a significant portion of their web space to showcasing studies that demonstrate the effectiveness of their product, for example, improving working memory, or generalizing to other activities (cf. Lumosity, 2013, “Completed research behind Lumosity”; Pearson, 2013, “Peer-reviewed research”). One could be forgiven, based on this evidence, for thinking that the ability to “retrain” the aging brain was already achieved, but in fact, most CT enterprises are involved with multiple ongoing studies examining further applications, generalizability, or other aspects of their products. Much of this research is done in conjunction with investigators at universities, or other institutions, such as pharmaceutical companies or retirement homes, that permit access to large groups of study participants and further validation of results (Lumosity, 2013, “Get involved overview”; Posit Science, 2013, “Partners”; Sharpbrains, 2013). While many groups are diversifying to new clinical populations via ongoing clinical trials, older users are major clientele of this software, and are aggressively marketed-to both in online literature, and other publications aimed at an older audience (Friedman et al., 2011; Millington, 2011; Vandenberg et al., 2012).

With this approach of neuroscience-backed marketing and strategic partnerships, corporate brain training initiatives’ involvement with the aging brain has been wildly successful, and continues to grow, several neurotechnology consulting groups, who have been monitoring CT’s development with great interest have recently posited that revenues are expected to climb to 6 billion dollars in the next decade, following a trend of exponential growth in coming years (Litinsky and Avila, 2009; Sharpbrains, 2012, “Executive summary”). This is remarkably good business for training companies, and other industries have taken note of both these profits and success stories from the field. Notably, exclusive alliances have been formed with several insurance companies such as Penn Treaty and Humanacare, and promoted as a way for policyholders to “actively address their concerns of developing dementia” (Brown, 2006; Posit Science, 2013, “Penn treaty first to offer brain fitness program”). Insurance companies are an important source of reification of medical knowledge, and have historically played an instrumental role in development of new medical categories (Brown, 1995; Rosenberg, 2002), and they are not the only way in which CT companies have expanded. Despite the fact that CT strategies are not recognized as a formal medical intervention for cognitive changes in later life, the pharmaceutical company Bayer has recently partnered with CT group CogniFit, hoping to develop training-based treatment for several neurological conditions (Bayer de Mexico, 2013). Additionally, CT centers are popping up in a variety of settings serving older adults, from hospitals (Baycrest, 2013) and more than 400 long-term care facilities in the US, Canada, and Europe (Fernandez, 2008; Litinsky and Avila, 2009), and being marketed directly to healthcare professionals to enrich their clients’ lives (Dakim, Inc, 2013, “Dakim BrainFitness software, professional”; Pearson, 2013, “Healthcare professionals”). Finally, interest in CT appears to have overflowed from purely biomedical frames of discussion to popular media (Friedman et al., 2011; Vandenberg et al., 2012), evidenced by a slew of recent publication in lay journals such as The Economist, New Yorker and The Wall Street Journal, exploring the new reach of CT technologies in the “new aging brain” (cf. Marx, 2013; The Economist, 2013; Reddy, 2013).

## Biocapital and bio-quandaries: definition of normal aging

At the heart of this hype and excitement lies what Franklin (2003); Sunder Rajan (2003, 2006) and others have termed “biocapital”. This way of understanding emerging technologies, the latter author writes, represents the fusion of biomedical innovation with profit-driven enterprise, and may thus be studied through the making and marketing of biological entities. (Sunder Rajan, 2003, 2006; Birch and Tyfield, 2013). Furthermore, biological material may itself become a player in networks of Foucauldian political and economic power, and alter individual subjectivities of participants in said networks (Foucault, 1978; Rose, 2007; Helmreich, 2008). While originally studied through ethnography in relation to physical biological matter such as DNA samples (Sunder Rajan, 2003), the rise of neuroscience-backed technologies has given rise to new blends of biocapital focused on the human mind and brain. Notably, Nikolas Rose and others have extensively written about this expansion, particularly with respect to neuropharmaceuticals and the way that they shape norms, ideas, and framing of human identity (Rose and Novas, 2003; Rose and Abi-Rached, 2013). While the biovalue created by CT in the form of a brain that is “sharp, healthy and young” (Brain Center International, 2014, “The ultimate brain fitness program”) is perhaps more abstract than a blood test or a gene chip, its development still parallels that of earlier medical technologies within biocapital. Further, the type of identity created within this process carries with it many of the risks and ethical quandaries found in previous profit-driven approaches entangled with the human mind and brain (Rose and Novas, 2003; Davies, 2010; Pitts-Taylor, 2010).

How then, may we study the rise of CT through with the tools of biocapital, and best conceptualize its interactions with the neuroscience of the aging brain? To this end, we begin by making a close investigation of the scientific claims underlying CT’s promises, and how they shape a new vision of aging. Despite their formidable successes in recent years, the approach of refining the aging brain through training has come under intense scrutiny. Naturally, a major source of debate has been whether CT programs, whether for young or old, live up to their scientific claims. In the realm of cognitive malleability in the aging brain, several meta-analyses and reviews have found evidence to suggest that training programs, indeed, prove an effective and promising avenue of intervention in older adults (Eschen, 2012; Kueider et al., 2012; Reijnders et al., 2013). On the other hand, several other works have found insufficient evidence to CT’s claims to effectiveness (Papp et al., 2009; Martin et al., 2011) or transfer to other domains (Noack et al., 2009; Buitenweg et al., 2012). Even with strong methodology and results, rubrics for defining success and persistence of effects differ significantly, which, coupled with assurance of ethical conduct makes large-scale validation and follow-ups difficult (Rabipour and Raz, 2012; Jak et al., 2013). It is beyond the scope of this paper to make definitive critiques about the efficacy of the vast diversity of products and approaches on the market (which are followed in more detail from the above-cited articles and meta-analyses), except to say that this high level of scientific scrutiny appears to continue unabated. With increasing tendencies toward biomedical validation, however, it is likely that most companies will continue to engage in this kind of dialogue. Ideally, ongoing studies and further affirmation will foster public interest in more research, new and different paradigms, and extension of ongoing clinical trials in new populations.

Yet, there are still a number of crucial questions to be examined about the entanglement of CT programs’ framing of aging and plasticity in ongoing programs. Quite apart from debates on the nature of late-life plasticity, the process of growing older is hardly a unitary concept or scientific fact. If neuroscience can be mobilized by training to remedy the aging process, which methodological and theoretical assumptions are propagated and emphasized in its vision of the process of growing older? At its simplest, CT is touted as a new way to slow changes associated with normal aging, and return healthy older adults to a level of function more comparable to “younger” brains. This idea is deceptively simple, and already implicates a wide variety of social, cultural and neuroscientific norms in creating “a rejuvenated brain that acts like 10 or more years ago” (Brain Center International, 2013, “Balance your brain”). Viewed even at the most basic neurobiological level, the changes in the aging brain that CT presumably remedies or reverses are extremely difficult to define, no less measure (Belleville and Bherer, 2012; Thomas and Baker, 2012). Research on neurological correlates of non-pathological aging is not a large field, but in recent years, evidence seems to be converging on a number of factors that change with advancing years in the brain. Structural changes to gray- and white matter occur, as well as a shift to more diffuse patterns of BOLD activation, and reductions in several populations of neurotransmitters have all been consistently observed (Raz and Rodrigue, 2006; Grady, 2008; Juraska and Lowry, 2012). These factors form the basis for current theories of plasticity in aging (Cabeza et al., 2004; Greenwood, 2007; Grady, 2012). Critically, due to the diversity of disciplines involved in the study of these factors, it unclear what relationship, if any, these findings possess to one-another, and a unified neurological vision of aging is far from complete. Further, it is not yet known whether these changes signal a fundamental underlying process, the net effect of environmental stressors on cellular health, or plastic changes to adapt to other forms of bodily degeneration (i.e., peripheral loss of hearing or vision capability).

It may be argued that as long as CT makes a tangible, positive difference in the lives of its trainees, this level of reductionism and exactitude in means of action is not necessary. Indeed, in extant product literature, the biological correlates of slowing cognition in normal aging are not discussed, save for general statements about the brain using much slower processing speed in day-to-day situations (cf. Sternberg, 2012; Posit Science, 2013, “Memory Lapses”), or how increased stimulation-based connectivity appears to be protective (HAPPYneuron., 2013, “Efficiency of brain training”; Pearson, 2013, “Brain fitness and brain training”). However, as CT operations move toward increasing medical validation and prescriptive use of their paradigms (as evidenced by increasing cooperation with insurance companies, the pharmaceutical industry and healthcare providers), defining more concrete routes of action will be critical, and must hold together with consistent data about qualifying the “ideal” trainee. If a given product or program is advertised to work on the “normally aging brain”, then substantial neurobiological delimiting factors will need to be elaborated-on, and form a basis for a better understanding of what does- and does not- accompany this heterogeneous collection of neurobiological processes.

In the absence of this knowledge, operationalizing “normalcy” in aging at a clinical/population level via more indirect measures presents a number of large challenges that are also absent from CT product interpretations of aging. Today, the clinical version of healthy cognitive function is mainly determined by test batteries, with “healthy” aging defined as better-than-cutoff performance on a series of testing criteria including The Mini-Mental State Exam, Abbreviated Mental Test or Mini-Cog, with more conclusive pathological diagnosis and monitoring made possible via in-depth neuropsychological test batteries and assessment of impairment in daily life (Woodford and George, 2007; Depp et al., 2012; Galvin and Sadowsky, 2012). These tools have helped researcher and clinicians to converge on a number of functions that typically deteriorate with age, most prominently difficulties with working memory, attention, and visuospatial accuracy (Cabeza et al., 2004; Reuter-Lorenz and Park, 2010; Grady, 2012). However, it has also been well-documented that aging populations demonstrate remarkable intra-group variance, which makes creating applicable “points of reference” for good cognition challenging. At one end of the spectrum are the “super normals”—individuals who outperform age-matched (and sometimes even younger) controls on most tests of cognitive function, and at the other, individuals who have been severely affected by cognitive decline at a relatively young age (Ylikoski et al., 1999; Ardila, 2007). Some investigators have even found evidence to suggest that cognitive decline begins in the early 30s, further complicating “boundary” conditions for defining change across the lifecourse (Salthouse, 2009a,b). Therefore, this category formation, a process already fraught with complex social influences and meaning (Young, 1995; Hacking, 2007; Slaby and Choudhury, 2012), is complicated by wide biological variance.

## Illness, health, and indeterminacy: explanation of age-related cognitive impairment and other pathology

Thus, while historical definitions of aging in Europe and North America relied on a clear dichotomy between externalized pathology and lack thereof (Katz, 1996, 2006; Jones and Higgs, 2010), today a scientific consensus is not so clear. Yet this perspective still appears to be employed in CT explanations of what one should expect in aging. What about other perspectives in this problem in the lived experiences of older adults, and rooted in traditions in the humanities and social sciences? To this end, our understandings of normal aging (and study thereof in CT) owes a significant debt to a number of authors writing in science and technology studies and anthropology of medicine. Notably, work by Margaret Lock has explored how tacit culturally-informed meanings interact with scientific discourse framing aging and biological changes (Lock, 1993, 2013). This spirit, guided by other important works by authors such as Lawrence Cohen on cultural politics of senility have helped shape a space for an anthropology of “normal aging” as a phenomenon irretrievably culture-bound, and framed by narrative, expectation, and behavior (Cohen, 1994, 2000). Sociological inquiry, too, draws from this model, but also emphasizes a whole-lifecourse perspective, where normality in aging is shaped by institutions and inequalities (Dannefer, 2011). As such, “normal aging” is a cultural construct deeply instilled with social values of autonomy and productivity (Jones and Higgs, 2010; Pickard, 2011). Drawing from work in both of these academic traditions, the next section of this paper will examine how CT engages with these differing definitions of aging, and employs neuroplasticity therein. Further, these processes will be examined in the light of diagnostic complications.

The most common understanding of the definition of “normal aging” in public discourse, and the one seemingly favored by CT enterprises, hearkens most back to a historical binary state, delineated by boundaries of recognized neuropathology. In this sense, one becomes “old” at a somewhat arbitrary age late in working life, but remains normal until signs of disease processes are evident in day-to-day actions. Heterogeneous age-associated changes, therefore, are largely framed in most forms of public discourse (including these sites), in terms of deterioration leading ultimately to pathology (cf. Cognifit, 2013, “Alzheimer’s”; HAPPYneuron., 2013, “Cognitive decline and brain training”; Posit Science, 2013, “Memory lapses”). Partially through the work of lobby groups and increasing public awareness, fears about progression to this type of unhealthy aging is most instinctively associated with Alzheimer’s disease (AD) and related dementias (Moreira, 2009b; Lock, 2013). In recent years, sobering reports on the prevalence and economic burden of AD have been widely published, including several by Alzheimer’s Disease International, The European Union and other international policy groups, illustrating and emphasizing the grave and immediate need for education and treatment (Council of the European Union, 2008; Alzheimer’s Disease International, 2009). Rising public consciousness about AD has also certainly played in role in the shaping of CT literature targeting older clients. Here, “healthy” cognitive functioning is generally defined as being a factor in daily annoyances, while the terrors of dementia or other diseases are equated to a loss of mental control and selfhood. These fears are strongly broadcast, occupying a central position in product promotion (cf. Dakim, Inc, 2013, “Give a loved one the mental stimulation they need”; Mind Evolve, 2013, “Improve memory and brain health”; Vandenberg et al., 2012). By promoting dementia-related anxieties, most companies frame age-related cognitive changes as a precursor to or emerging symptom of cognitive dementia, making statements such as “It is proven that cognitive abilities decline with time if nothing is done to prevent it” (Cognifit, 2013, “Mind Exercises”). In this light, elucidating whether training can prevent small cognitive changes from progressing into pathological processes is a major topic of research still being explored. Some studies have shown modest improvements and delaying of symptoms for individuals with AD or MCI (Belleville, 2008; Valenzuela and Sachdev, 2009; Herrera et al., 2012), but others have failed to find an effect (Jean et al., 2010; Unverzagt et al., 2012). Nonetheless, training is touted as playing an instrumental role in halting disease progression on several sites (Dakim, Inc, 2013, “The science behind Dakim Brain Fitness”; Brain Center International, 2013, “Balance your brain”), further helping to reinforce ideas about normality and pathology-related deviation.

How, then, could CT use these expectations to actively define a space for prescriptive action in the aging brain? Even guiding a seemingly clear dichotomy between to-be-expected decline and pathology is party to historical and contemporary debates surrounding category boundaries in old age (Gaines and Whitehouse, 2006; Whitehouse and George, 2011; Williams et al., 2012). Despite researchers’ best efforts, reliable diagnostic markers for AD and other dementias have proved elusive, and broadening pre-existing disease categories to include wider diagnostic criteria and extend the reach of treatment has proved highly contentious. A relevant example comes from the recent development of a disease category for Mild Cognitive Impairment, non-specific cognitive changes usually linked to progression of other conditions like AD (Golomb et al., 2004; Gauthier et al., 2006). This move has caused controversy both within and outside of the medical community (Graham and Ritchie, 2006; Whitehouse and Moody, 2006), and heightened awareness of the complexities and seriousness of cognitive decline at all stages in older adults. For brain training companies, drawing from current public discourse on dementia is a way to help consumers appreciate the gravity of maintaining and understanding cognitive health across the lifecourse, and perhaps take “protective” steps that they might not otherwise be aware of. Even if, as one group writes, “because of your brains (sic) special plastic powers, you have a much better chance of recovering [from] disease” (Dakim, Inc, 2009, “Neuroplasticity”), contrasting pathology and (guided) plasticity cannot satisfactorily delineate normal from abnormal aging. If training enterprises are to make dementia-based anxieties a foundational part of their message and product claims, large bodies of research examining the subtleties of brain-based decline in old age may be glossed-over, not to mention the complex interaction of disease processes with environmental, social and cultural factors. This makes CT’s version of the aging brain a very limited and limiting frame for viewing the complexities of the aging process.

The promise of a non-invasive, accessible way to prevent dementia has obvious appeal, and perhaps that reason alone has driven the bulk of CT’s success. However, this presents a challenging version of biocapital, where the value of a product is deeply entangled with hope and expectation surrounding its claims to future success. Study of exactly this phenomenon has grown in recent years to a diverse field studying scientific futures or the “sociology of expectations”, where the promises and predictions surrounding a technology drive change at both an economic and social level (cf. Novas, 2006; Pollock and Williams, 2010; Tutton, 2011). In the example of scientific futures in anti-aging technologies, it has previously been noted that fears about growing older are employed to propel research and legitimation in a disputed field (Mykytyn, 2006). To put it more bluntly, new technologies may employ what Joe Dumit has termed a “venture science”, where expectations are employed to simultaneously create new biomedical knowledge and economic growth (Dumit, unpublished paper). In the case of CT, plasticity is promised as an antidote for aging, and a way to “regain the brain power they enjoyed 10 years before” (Brain Center International, 2014, “Neuroplasticity”). However, these promises reach beyond a simple prophylactic framework, and encompass wider area of concern and critical analysis surrounding discourses found in their perspective on the normally aging individual and brain.

## Situating plasticity in a social world: sociocultural factors involved in shaping brain health

Just as the development of MCI as a framework for understanding cognitive changes has attracted attention criticism from scholars in the natural and social sciences (cf. Graham and Ritchie, 2006; Hyman, 2006; Graham, 2008), the framing of the “normally” aging brain as a homogenous zone of neurotechnology-backed intervention may become a problematic way to understand and interact with that category. The relationship between concepts of aging and the biosciences has never been truly straightforward, but in North America and Europe, individuals’ lives in the “third age” after retirement have in the past century increasingly become influenced by ideas stemming from advances in science and medicine (Kaufman, 1994; Katz, 1996; Powell, 2006). In 1989, Estes and Binney (1989) coined the phrase “biomedicalization of aging” to describe what they termed “the social construction […] and praxis of aging as a medical problem”. This process, they write, is thought to create a unimodal understanding and method for dealing with of the aging process, excluding social or cultural interpretations or forms of interaction. The biomedical tradition already maintains a powerful influence on most pathological processes in old age such as preventative lifestyle practices or views about death and dying, but growing interest in anti-aging technologies has promoted a view of the typically aging body according to a medical gaze and focus on pathology (Rose, 2001; Kaufman et al., 2004; Mykytyn, 2006; Powell, 2006). More recently, several authors have posited that these trends are employing widening interest in neuroscience to draw nearer to the mind and brain (Rose, 2007; Katz and Peters, 2008; Millington, 2011; Williams et al., 2012).

Evidently, therefore, biomedical control is neither a new phenomenon nor area of study. Neither is the idea that under the auspices of medical-scientific knowledge, new categories of social life may be created and altered. Since Paul Rabinow’s seminal work on “biosociality”, or the formation of new networks united by biological qualities (Rabinow, 1996), many anthropologists and sociologists have explored shifts to these new forms of identity and interaction. From ethnographic field work with carriers of susceptibility genes (cf. Gibbon, 2007; Lock, 2013) to new conceptions of biovalue caught up with patient networks and activism (cf. Novas, 2006), it has been acknowledged that science and medicine can cause profound shifts in social life and organization. This is especially evident in neuroscience-related disciplines, where biological explanations of the brain and mind appear to deeply influence social environments and ideas about personhood and human nature. In the case of mental illness, authors such as Joe Dumit and Emily Martin have written extensively on both the problematic and liberating views held by individuals within these neuro-centric networks (Dumit, 2004; Martin, 2009). Neurological selfhood has also been explored in relation to new technologies, most notably, the rise of neuro-pharmaceuticals and their consequences for defining moral obligations and identity (Rose, 2007; Rose and Abi-Rached, 2013). While CT can draw heavily from these previous descriptions, particularly with respect to the role of technology in shaping lifestyle, it differs in several key aspects. First, CT seeks to situate itself in a historically (and contemporarily) liminal category, where boundaries of pathology are unclear. This means that there is a strong impetus to define a target population, and create a prescriptive frame for action. While other forms of medical intervention have formed communities based on “embodied risk”, or future susceptibility to a condition (Kavanagh and Broom, 1998; Lock and Nguyen, 2010), CT seeks to do the opposite, by creating a framework based on *normality*. The key to this angle is plasticity, which unites prospective consumers by the possession of an untapped resource that can strengthen their degree of normality. Thus, an *inherent* quality of the human brain is monopolized to create a fresh, homogenous take on how to age well, and promise distancing from the threat of abnormal pathology. As we will see below, although this message has many liberating characteristics, it ultimately carries a weighty moral comment that will be explored below.

Additionally, it is still important to remember that CT programs for older adults differ from many previously-studied agents in the creation of new networks in that it cannot be formally prescribed as a medical intervention. Therefore, its liminal phase outside of formal interventions represents both a challenge for study, and a boon to the companies themselves. Several companies use this to their advantage, stressing a “Non-invasive […] drug free” approach (Posit Science, 2013, “How brain training works”), differentiating themselves from non-organic methods which “artificially and temporarily [alter] the way your brain works” (Pearson, 2013, “Cogmed is based on a research breakthrough”). With or without official sanction from biomedical authorities, their technologies appear to have been embraced by consumers, pharmaceutical and health insurance companies and become linked with a growing social impetus to intervene in and potentially control aging. As much as this drive may be considered a scientific endeavor to learn and repair the aging brain, it is entangled with ideas about aging that stem from social spheres. These, no less than biochemical justifications validate and strengthen CT companies’ vision of the again brain, and become enmeshed with neuroscientific explanations of human aging as concrete, “treatable” facts. In this light, it is fundamental to understand and perhaps challenge which social versions of aging are appropriated in CT literature, and just how they interact to form a vision of aging so palatable to industry and the public.

A brief glance at most neuroscience-based product literature demonstrates an interesting mix of ideas from both biomedically- and socially-informed spheres of aging. To review, neurochemical aging is employed by almost all groups through messages about employing training with a view to prevent dementia and other pathological processes. According to this approach, “normalcy” is concrete, all-inclusive, and delineated by scientific knowledge. It is a space where, sooner or later, most aging adults will find themselves prior to descent into illness, and where they could stand to benefit from training approaches. “Staving off and/or slowing down dementia”, explains one product website, “…is therefore the obvious reason for seniors to engage in a dedicated brain fitness program” (Dakim, Inc, 2013, “The science behind Dakim Brain Fitness”). Employing plasticity is the key to this approach, and will allow the user to slow down the processes of aging, and potentially prevent pathology. Thus, aging is a unitary, degenerative process which may only be altered by appropriate, technology-backed intervention and training. However, this singular and rather dark vision is in stark contrast with more positive interpretations strongly reminiscent of literature from seniors’ rights groups, biogerontologists, and others decrying the image of older adults as a homogenous, impaired group (Coupland and Coupland, 1993; Blaikie, 1999). For example, one company speaks of “successful aging” (Brain Center International, 2013, “Science’s way to brain health”), while another emphasizes the brain’s ability to “grow and change at any point in the lifetime” (Lumosity, 2013, “Your brain is amazing”). In other cases, using training to engage plasticity creates “successful agers” who are “optimistic, resilient in the face of life’s changes, and have a well developed sense of control. And they’re constantly stimulating their cognitive faculties” (Brain Center International, 2014, “Successful Brain Aging”). This, finally, is where CT’s heritage of experience-dependent plasticity comes into full play, in demonstrating the brain’s marvelous capacity to be shaped by the conscientious user.

While this approach presents a flexible and *self-defined* view of aging, it is no less important to explore in the light of its framing of plasticity and normalcy. Strategies based on self-investment through improvement of health have increasingly become an area of investigation and critique in the past decades, and succinctly categorized by Michel Foucault as “technologies of the self”. This process, he writes, “permits individuals by their own means or with the help of others a certain number of operations on their own bodies […] so as to transform themselves in order to attain a certain state of happiness, purity, wisdom, perfection, or immortality” (Foucault, 1988). Already well-noted in the literature surrounding “lifestyle” or “active living” interventions aimed at the (aging) body (Katz, 2000, 2006; Millington, 2011), this type of will to health is somewhat newer to the mind and brain (Rose, 2005; Friedman et al., 2011; Williams et al., 2012). Experience-dependent plasticity may assume this role as a “pragmatic, holistic, and effective” avenue for change in the brain in CT literature (Pearson, 2013, “Cogmed is based on a research breakthrough”), even if knowledge about age-related pathology appears to still underlie the fundamental impetus to use these training programs. The aging brain, therefore, is framed as a new theatre of action, in which only plasticity-based interventions, when “thoughtfully and methodically explored” via training motivated and targeted scientific interventions will perform the maintenance and repair necessary to “remember more, think faster, and *achieve your full potential*” (Lumosity, 2013, emphasis in original, “Your brain is amazing”) without the worry of disease.

In the view of scientists, clinicians and policy-makers, this level of interest in (and seriousness accorded to) cognitive health is a fundamental factor in maintaining a healthy aging population (cf. Depp et al., 2012; World Health Organization, 2012). Indeed, should the most optimistic promises of CT turn out to be true, then caring for cognitive health in this way would have major beneficial effects. Yet, the ways in which this message about the promise of changeability is framed and promoted in the development of CT still needs to be appraised carefully, with particular attention to the ways in which neuroscience is used to define, delimit and remedy the complex collection of processes through which the process of aging manifests itself. In this case, experience-dependent plasticity is used to promote a highly individualized relationship between an older individual and their brain, where only motivation and body work on the part of the former can save the latter. And the stakes are high. Several companies equate age-related cognitive decline to losing your “Life Story”, (Vivity Labs, 2013, “Fit Brains FAQ”), or a brain that is “ever present, ever listening, ever learning, obeying our every command, solving our every problem, attending to our emotions and wishes, remembering our joys and sorrows, […] our best friend” (Cognifit, 2013, “Train your brain seriously”). Could this be what Rose and Novas (2003) have described as a “moral economy of hope”, where the promises of new technology both augment its biocapital and engender responsibility on the part of the user?

The predictive power of these claims aside, this level of entanglement between health and identity in anti-aging technology has been identified by numerous authors and activists as creating a problematic “will to health”. This situation places an individual in a narrow relationship with their body and promotes a moral and ethical imperative to healthful action. Further, it may subsequently neglect important cultural and social factors and inequalities that shape the user’s lifeworld (Estes and Binney, 1989; Vincent, 2006; Higgs et al., 2009). This is especially important for a body of work relying on experience-dependant plasticity, about which more and more is learned every day about how neurological malleability is acted-on by a variety of extrinsic forces. Since findings from early epidemiological data from the 1920s, it has largely been accepted that several social factors are an important driving force for the health of adults in later life, including social support, socioeconomic status and mobility (Smith and Kington, 1997; Ó Luanaigh and Lawlor, 2008), and several of these factors have also been associated with structural diversity in the adult and aging brain (Fotenos et al., 2008; Krishnadas et al., 2013). Even an individual’s subjective beliefs about aging and cognition have been shown to be a major factor in cognitive performance (Seeman et al., 1996; Hertzog and Hultsch, 2000; Cook and Masrirske, 2006). In this way, CT is potentially placed in a position to both mold and “repair” an individual’s self-concept, creating and shaping identities linked to understandings of their brain and mind (Ortega and Vidal, 2007; Vidal, 2009). Each of these potentially plasticity-inducing factors exists within a complex web of interaction, and as such, may not be seen as purely or unidirectionally causative (Kramer et al., 2004; Hackman et al., 2010 ). Yet, via mechanisms that are not fully understood, they appear to engage with the malleable brain in ways that shape cognitive health in later life and that *may or may not* ultimately be changed by training. The biocapital of CT, therefore, appears to hinge on a type of scientific future where experience-dependent plasticity is a singular commodity rather than all-encompassing feature of lived experience.

## Moving forward with cognitive training

Taken together, ideas surrounding the potential for operationalizing plasticity in the “normally” aging brain form a complex dialogue, drawing from a variety of sources to promote individual responsibility in exercising the mind and staving off cognitive decline. While the possibility of intervention is a powerful message, it constructs the aging mind as a zone of tension between discourses on the nature of aging, personalized possibility of intervention, and a brain only selectively permeable to experience-dependent plasticity. The largely positive messages of CT literature trumpet the advances of contemporary scientific understanding of aging, and promise tangible improvements and long-term effects of exercising the mind as a muscle. While such intervention in normal aging is still a fairly young concept in both scientific and public spheres, it is a powerful one, opening new possibilities for dealing with cognitive decline and redefining the aging mind positively as a flexible entity. It is not yet known whether this vision of the aging mind and brain will be ultimately liberating or constraining, or how training will evolve to fit an increasingly sophisticated understanding of the constant negotiation of social, cultural, and environmental factors shaping cognitive function in later years. At present, CT paradigms that target the aging brain represent an immense opportunity, not only in terms of scientific progress, but also in capturing public consciousness about cutting-edge neuroscience and placing it in a very real, very immediate context, which sooner or later, most individuals will experience. Therefore, now more than ever, it is crucial to move forward with integrative research to understand the dimensions and limitations of this new paradigm for understanding the aging mind.

Moving forward in research and treatment with CT absolutely and necessarily requires ongoing verification and challenge to existing training paradigms and constant monitoring to assure that viable strategies, adequate control groups and unbiased research are actively promoted (Elias and Wagster, 2007; Rabipour and Raz, 2012; Thomas and Baker, 2012). In addition, there is great opportunity to engage in thoughtful, holistic research to better understand how the concept of a malleable brain gains from, and contributes to ongoing discourse in both the social and natural sciences. If the brain is truly as plastic and embedded in its sociocultural context as we believe, then it represents the perfect milieu for interdisciplinary research on aging. At one end of the spectrum, more basic neurobiological work must be done to more fully understand the basic processes involved in producing different ranges of function at different time-points in the lifecourse, and understand that the way which plasticity is mobilized to promote adaptation to ongoing needs of the organism may too be different. However, this research must recognize that due to the experience-dependent nature of the brain, aging can necessarily not be the same for all individuals, and therefore cannot be reduced by bookending with pathology. In this sense, the roles of biogerontology, medical sociology, and transcultural psychiatry are paramount, to explore the ways in which an individual’s environment selects for, protects against, or interacts with ongoing biological processes to produce cognitive change in later years. Perhaps more importantly, these disciplines, coupled with approaches from anthropological and philosophical traditions, can explore the ways in which understandings of aging impact an individual’s self-concept, and beliefs about lifecourse transformations and their effects on the brain and mind.

If CT can be used to create a space for research examining plasticity and embeddedness by looking *inward* to underlying processes, then it should equally be used in looking *outward* to a new vision of aging and change on individual, cultural, and social spheres. Using Hacking’s concept of “bio-looping”, through which cultural contexts of science interact with biomedical knowledge and actors in a reflexive process (Hacking, 1995, 2007; Choudhury et al., 2009), this new field of neuroscientific knowledge may be productively explored in respect to wider social processes. Though still new technology, it is important to examine which elements of this malleable aging brain get carried through to policy formation, or ways of promoting public health within the community. At the level of research and knowledge transfer, it will further be interesting to monitor how the successes and failures of CT are adopted or rejected by other forms of emerging neurotechnology. As a training approach that still straddles uneven territory between experimental work and wide-scale preventative medicine, many groups have an investment in CT, and the renegotiation of its status through positive messages from popular media and advertising will create interesting dynamics between stakeholders, scientists and the lay public. Will the idea of a brain that changes itself in late life have the power to alter these individuals’ self-concepts, of affect the process of identity formation? These, and many other questions could represent fruitful avenues for future research.

## Limitations and conclusion

As it stands this type of work is in its infancy, and the present paper cannot be said to explore all dimensions of the true ongoing dynamics of plasticity, CT, and new dimensions of aging. While using public information communicated by CT companies is an important first step, particularly with respect what potential consumers are likely to encounter in descriptions of late-life plasticity, this approach presents several limitations. In the future, we hope to expand our investigations to include first-hand research with participants involved in CT, the companies that produce them, and the various agencies that help communicate CT’s promises and complexities to the public. Thus, it is our hope to better expand on the goals and motivations of these players, and understand how they interact to create new knowledge about the brain. Time will reveal the qualities of adult neuroplasticity upheld by CT have the power to create new understandings of aging, and more research will undoubtedly add nuance to the story that this paper could not.

In conclusion, new understandings of neuroplasticity have helped shape a scientific and cultural space for change and reorganization in the aging brain, opening diverse possibilities for exploring treatment and enrichment. Molded through the influence of CT initiatives, it presents a framework where influences from biomedical and cultural spheres shape a vision of hope and challenge in understanding the aging brain as a zone for change and improvement, new discoveries in neuroscience can be mobilized to promote a vision of an active, healthy mind and brain. Though this framing of an individual as a biological and social entity bears room for analysis and critical thought, especially as CT enterprises further diversify and grow, it is one which represents an amazing space for integrative research in how the aging brain is shaped by a variety of entities, and ultimately, represents one of the most intellectually challenging spaces for contemporary natural and social sciences.

## Author contributions

Constance Holman researched and wrote the paper, Etienne de Villers-Sidani contributed to clinical and scientific aspects and wrote the paper.

## Conflict of interest statement

The authors declare that the research was conducted in the absence of any commercial or financial relationships that could be construed as a potential conflict of interest.
